# Luminal-Type Invasive Carcinoma in Association With Microglandular Adenosis/Atypical Microglandular Adenosis: A Case Report and Molecular Comparison

**DOI:** 10.7759/cureus.37198

**Published:** 2023-04-06

**Authors:** Couger Jaramillo, Ashley Nazario-Toole, Hui Xia, Thomas Adams, Michelle Josey

**Affiliations:** 1 Department of Pathology, Brooke Army Medical Center, San Antonio, USA; 2 Clinical Investigations and Research Support Laboratory, 59th Medical Wing, Joint Base San Antonio-Lackland, San Antonio, USA

**Keywords:** atypical microglandular adenosis (amga), breast cancer biology, luminal-type invasive carcinoma, microglandular adenosis associated carcinoma (mgaca), microglandular adenosis (mga)

## Abstract

Microglandular adenosis (MGA) is a proliferative breast lesion composed of small, uniform glands lacking a myoepithelial cell layer while still invested by the basement membrane. The glands percolate haphazardly through the breast parenchyma rather than maintaining a lobular architecture, typical of other forms of adenosis.MGA is a benign lesion though atypical forms have been well described, often in close association with carcinoma. MGA, atypical MGA (AMGA), and the vast majority of MGA-associated carcinomas (MGACA) are negative for estrogen receptor (ER), progesterone receptor (PR), and human epidermal growth factor 2 (HER2) by immunohistochemistry. In light of these findings and early molecular studies, MGA is hypothesized to represent a clonal process and nonobligate precursor of basal-type breast carcinomas. We present the case of a 58-year-old woman and the first published molecular comparison of a luminal-type invasive ductal carcinoma with its associated MGA/AMGA. Analysis of small nucleotide variants (SNVs) revealed that 63% of the SNVs identified in the MGA were present in the AMGA while only 10% of them were present in the MGACA, suggesting a direct relationship between MGA and AMGA but not MGA and MGACA.

## Introduction

Microglandular adenosis (MGA) is a rare proliferative breast lesion that has been reported in women over a wide age range (28 to 82 years old) with a mean age of diagnosis in the sixth decade of life [1,2]. No specific predisposing factors have been identified. MGA is often an incidental finding but may present as a mammographic density or calcifications [2-6]. MGA is often inconspicuous on gross examination though it has been described as a poorly defined nodularity or mass. A spectrum of morphologies of MGA has been described, from the usual MGA lacking atypia to atypical MGA (AMGA) to carcinoma arising in association with MGA (MGACA) [2]. On the basis of shared morphological, immunohistochemical, and molecular features, it has been suggested that MGA is a nonobligate precursor to triple-negative breast cancer with increasing architectural and morphologic complexity reflecting the acquisition of increased genetic alterations [7-11].

Histologically, MGA is composed of small, uniform glands lacking a myoepithelial cell layer [12,13]. The glands are round, glandular structures with open lumina, lined by a single layer of bland, flat to cuboidal cells, and infiltrate haphazardly through the breast parenchyma, evoking no desmoplastic stromal reaction [2,14]. Periodic acid-Schiff positive luminal secretions and/or calcifications are often present. The glands are invested by a basement membrane, which may be demonstrated by electron microscopy or with collagen IV or laminin immunohistochemical (IHC) stains [15]. MGA is characterized by an ER negative, PR negative, HER2 negative, CK5/6 negative, CK8/18 positive, epidermal growth factor positive, and S100 positive immunophenotype [2,10,11,16,17]. The differential diagnosis of MGA typically includes adenosis, tubular carcinoma, and/or acinic cell carcinoma [1,12,18]. Atypical microglandular adenosis (AMGA) is characterized by increasing architectural complexity to include luminal bridge formation and microcribiform nests, fused, elongated tubules, solid nests, and single cells. AMGA should also show cellular stratification, mild to moderate cytologic atypia, and obvious mitoses [2,17]. Additionally, Ki-67 and p53 show relatively increased positivity from MGA to AMGA [2,16,17]. Invasive carcinoma arising in MGA has been reported in up to 20-30% of MGA cases [2,16,17]. MGACA are phenotypically diverse and have been reported in subtypes including adenoid cystic carcinoma and carcinomas with basaloid, secretory, squamous, chondroid, and chondromyxoid features [10,19-21]. However, MGACA is most often high-grade invasive ductal carcinoma with a triple negative and S100 positive immunophenotype [16].

MGA, AMGA, and MGACA are genetically heterogeneous entities, with the most common nonsynonymous mutation reported to be shared when they co-occur being found within the TP53 gene [2,22,23]. Additionally, somatic mutations in genes involved in the P13K/Akt/mammalian target of rapamycin (mTOR) pathway (PAM) and the tyrosine kinase receptor signaling-related genes FGFR2 and ERBB3 have been identified in TP53-mutation negative cases [11]. The PAM pathway is crucial to cellular growth/synthesis; growth factors binding to PI3K leads to a series of phosphorylation steps ultimately activating AKT, the central mediator of the pathway, which in turn activates mTOR to stimulate growth and synthesis [24]. Alterations of the PAM pathway are implicated in many cancer types, including as many as 70% of breast cancers (most commonly ER-positive), showing pathway activation [24]. Alterations have also been identified in hormone receptor-negative breast cancer. Mutations in PAM pathway genes, PTEN, PIK3CA, and INPP4B, were identified in TP53-mutation negative MGA/AMGA [11,24,25]. FGFR2 encodes fibroblast growth factor receptors in epithelial (FGFR2b) and mesenchymal (FGFR2c) cells with cytoplasmic tyrosine kinase domains involved in the mitogen-activated protein kinase, PI3K, protein kinase C, and calmodulin-calcineurin-NFAT signaling cascades [26]. In mouse models of FGFR2, activation enhances branch morphogenesis in mammary glands and predisposes to tumorigenesis by promoting epithelial-mesenchymal transition and negatively regulating BRCA1 [27]. Copy number variations are another form of genetic alterations found in MGA/AMGA/MGACA and described previously in the literature [10,22,23,28]. According to one case series, a median of 20.3% of the genome in a pooled sample of MGA, AMGA, and MGACA harbor copy number variants with an increase in the median number [22]. Moreover, a stepwise increase in copy number variants from MGA to AMGA to MGACA has been reported [11,22]. Recurrent gains found in MGA/AMGA include those in 1q, 2q, 7p, 7q, and 8q while recurrent losses include those in 1p, 8p, 17p, 5q 14q, 16q, and 17q [11,22]. Additional recurrent changes reported in AMGA include gains of 6p and losses of 10q [22]. Amplifications of the MYC gene (8q24.21) and DEPTOR (8q24.12) have been reported as well [10,11]. A subset of MGAs and AMGAs lacking copy number variants has also been described [10,22].

Rare case reports of hormone receptor-positive MGACA are documented in the literature, however, none have undergone molecular characterization [16,17,28,29]. The current study presents a case report and the first molecular characterization of hormone receptor-positive invasive ductal carcinoma arising in the setting of MGA and AMGA.

## Case presentation

**Figure 1:**
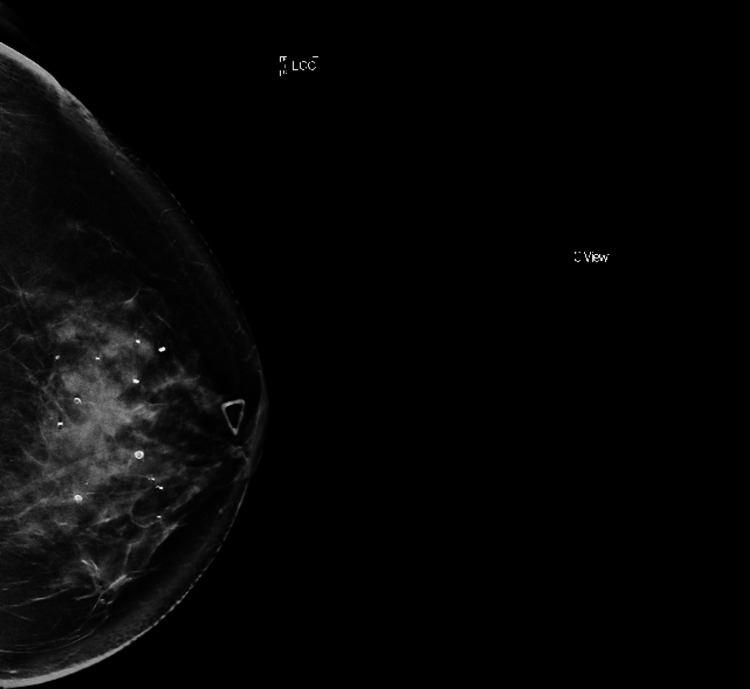
Diagnostic mammogram revealed a 2.3 x 1.2 x 2.5 cm irregular, hypodense mass with spiculated margins in the left breast

A 58-year-old nulliparous female presented to our institution with a new, palpable left breast mass, first noticed four days prior. Her medical history was significant for menarche at age 15, menopause at age 50, a 14-year history of birth control use, and a family history of breast cancer in a maternal aunt (62 years old at the time of diagnosis). At the time of presentation, the patient denied breast pain, dimpling, retraction, or discharge. Physical examination was notable for a 4 x 3 cm palpable left breast mass at the 12:00 position, approximately 2 cm from the nipple involving both upper quadrants with unremarkable overlying skin. A diagnostic mammogram revealed a 1.3 cm oval mass in the left breast with obscured margins favored to be benign (BI-RADS (Breast Imaging Reporting & Data System) Category 3). On follow-up seven months later, a 2.3 x 1.2 x 2.5 cm irregular, hypodense mass with spiculated margins located 2 cm from the nipple in the 12:00 position classified as BI-RADS Category 5 was identified on a diagnostic mammogram (Figure 1). No axillary or internal mammary chain lymphadenopathy was appreciated on imaging. The patient underwent an ultrasound-guided needle core biopsy that revealed moderately differentiated luminal-type invasive ductal carcinoma and a background of MGA. Notably, cases of hormone receptor-positive invasive ductal carcinoma arising in the setting of MGA are exceedingly rare, limited to a handful of case reports in the literature. She was clinically staged as a cT2, cN0, M0 and was referred for breast-conserving therapy. The patient underwent a left lumpectomy with sentinel lymph node biopsy with clear margins for her invasive disease, but extensive AMGA was present at the superior-medial margin. She was subsequently treated with adjuvant radiation therapy and maintenance anastrozole. The patient remains radiographically free of disease at the time of this publication, 18 months post-surgery.

## Discussion

Gross examination

The biopsy specimen consisted of a 2.1 x 1.2 x 0.3 cm aggregate of unremarkable white-tan soft tissue. The lumpectomy specimen demonstrated a single 3.5 x 2.1 x 1.6 cm, firm, white-tan mass with ill-defined, irregular borders that appeared to extend to the anterior, medial, lateral, superior, and inferior margins grossly. An additional shave of the superior-medial margin was submitted at the time of surgery, which appeared to have a grossly unremarkable cut surface. Two grossly unremarkable lymph nodes measuring 1.5 x 0.7 x 0.5 cm and 1.2 x 1 x 0.8 cm were recovered from the sentinel lymph node biopsy specimen.

Histology results

**Figure 2:**
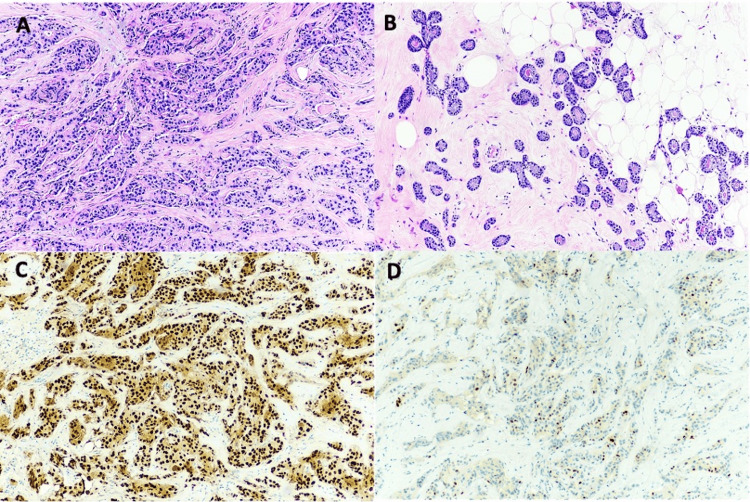
Ultrasound-guided core needle biopsy demonstrated moderately differentiated invasive ductal carcinoma (2a) in a background of small uniform glands lined by bland cuboidal cells with open lumina and intraluminal eosinophilic secretions scattered in the fibrous and fatty stroma consistent with MGA (2b). Immunohistochemistry was performed on representative sections and further characterized the invasive component as ER-positive (2c), PR-positive (2d), HER2-negative A-B: H&E stain, 10x objective lens. B-C: 10x objective lens

Microscopic evaluation of the biopsy specimen revealed moderately differentiated invasive ductal carcinoma measuring in a background of microglandular adenosis (Figure 2). The microglandular adenosis was characterized by well-formed uniform glands with eosinophilic luminal secretions scattered throughout the fibrous and fatty stroma. A preliminary Nottingham histologic score of 2 was assigned to the invasive component (3/3 tubule formation, 2/3 nuclear pleomorphism, 2/3 mitotic activity; total score=7/9). No lymphovascular invasion was identified on routine sections.

**Figure 3:**
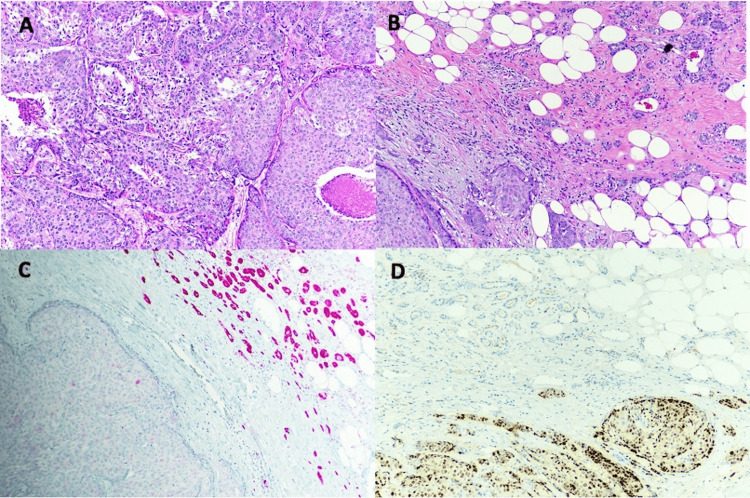
Lumpectomy specimen redemonstrated the moderately differentiated invasive ductal carcinoma (3a) but the adjacent MGA reflected increased architectural complexity and cytologic atypia most consistent with AMGA (3b). Immunohistochemistry was performed and is positive for S100 in the AMGA (3c) and PR in the invasive component (3d), clearly delineating the interface between the two morphologically distinct lesions A-B: H&E stain, 10x objective lens. B-C: 10x objective lens

Microscopic evaluation of the excision specimen showed a 3.5 cm, moderately differentiated invasive ductal carcinoma with a final Nottingham histologic grade of 2 (3/3 tubule formation, 2/3 nuclear pleomorphism, 2/3 mitotic activity; total score=7/9) (Figure 3). Directly adjacent to the invasive carcinoma were areas of MGA with classic morphology consistent with the biopsy specimen, as well as MGA with atypical features, including nuclear stratification, nuclear atypia, prominent nucleoli, increased mitoses, compressed lumina, elongated/fused glandular units, cribriform glands, and single-cell infiltration, consistent with AMGA. The final pTNM AJCC 8th Edition Pathologic Stage Classification assigned was pT2 pN1 mi(sn).

Immunohistochemistry results

Immunohistochemistry for prognostic markers was performed on the biopsy specimen and characterized the invasive component as ER-positive (91-100%, strong), PR-positive (51-60%, weak to moderate), and Her2-negative (2+ on IHC, negative by fluorescence in situ hybridization (FISH)), with a Ki-67 proliferation index of 20%, consistent with a luminal type (Figure 2). Lymphovascular spaces free of invasion were identified with D2-40 and CD34.

Immunohistochemistry for ER, PR, HER2, and Ki-67 was repeated on the final resection specimen, redemonstrating a luminal-type profile in the invasive component (estrogen receptor-positive (80-90% cells with positive nuclear staining with moderate average intensity), progesterone receptor positive (60-70% cells with positive nuclear staining with weak-moderate average intensity), HE2 negative (1+), and 20% Ki-67 positivity). Areas of MGA and AMGA demonstrated typical strong, diffuse S100 positivity and negative for ER and PR (Figure 3). Further, the MGA and AMGA areas were negative for myoepithelial cells by p63 and calponin. IHC for p53 was wild-type (patchy, weak staining) within the MGA, AMGA, and MGACA components. One of two submitted sentinel lymph nodes was involved in micrometastatic carcinoma, highlighted by IHC for pancytokeratin.

These histological and immunohistochemical findings are diagnostic of a rare case of luminal-type invasive ductal carcinoma arising in the setting of MGA/AMGA. To date, instances of hormone receptor-positive MGACA are limited to a handful of case reports in the literature (Table 1).

**Table 1:** Literature review of hormone-positive MGACA cases A comprehensive literature review of all case reports of luminal-type microglandular adenosis-associated carcinoma (MGACA) with patient demographic information, histologic subtype, and hormone status by immunohistochemistry organized by year of publication. MGACA: microglandular adenosis-associated carcinomas

Source/Year	Age/Sex	Histologic Type	ER	PR	HER2
James et al. 1993 [16]	Not reported	High grade ductal	Positive ("strong")	Positive ("strong")	Negative
James et al. 1993 [16]	Not reported	High grade ductal	Positive (intensity not reported)	Negative	Negative
Koenig et al. 2000 [17]	Not reported	Not reported	Positive (weak)	Negative	Not performed
Choi and Bae 2013 [29]	44F	Not reported	Positive (Allred score 3)	Negative	Negative
Damron et al. 2019 [28]	47F	Well-differentiated ductal	Positive (40%, 3+ intensity)	Positive (40%, 3+ intensity)	Negative (1+)

Sequencing results

**Figure 4:**
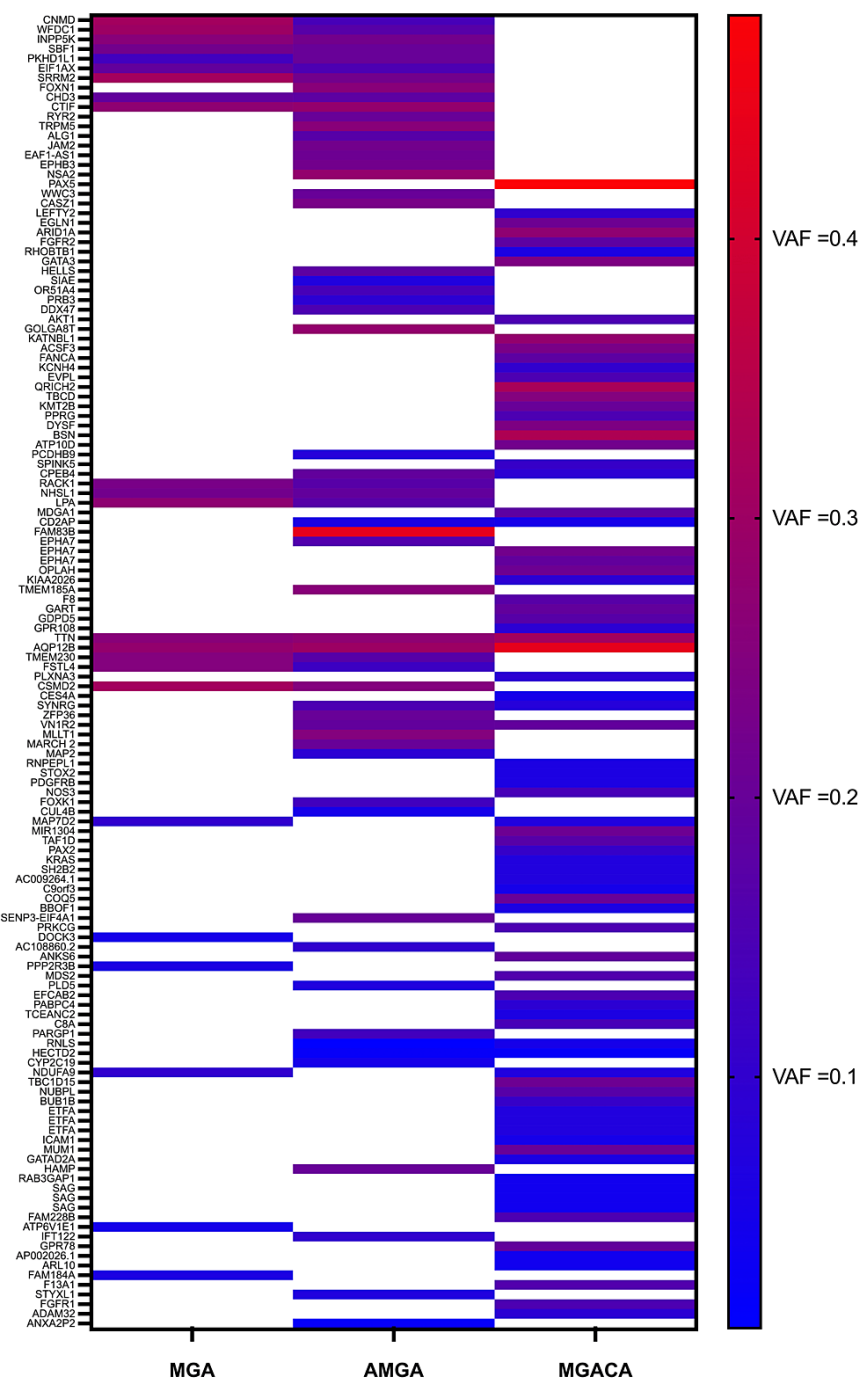
Heatmap of all single nucleotide variants identified by whole exome sequencing with variant allele frequency > 0.05 identified in the microglandular adenosis (MGA), atypical microglandular adenosis (AMGA), and MGA-associated carcinoma (MGACA) specimens organized by affected gene and morphological type

Twenty-seven single nucleotide variants (SNV) were identified in the MGA sample, 63 in the AMGA sample, and 78 in the MGACA (Figure 4). Only two SNVs were shared between all three distinct histologic entities (intronic variants in MIR1304 and TAF1D), neither of which have been previously described in MGA or breast cancer. Additionally, a third SNV that failed to reach the threshold for variant allele frequency of 0.05 in the AMGA sample was shared by all three histologic entities (C9orf3, see Supplementary Data in the Appendices). Seventeen of the 27 SNVs identified in the MGA were shared with the AMGA. Six of the 63 SNVs identified in the AMGA were also present in the MGACA. Notably, two different nonsynonymous variants associated with breast cancer were identified in the AMGA (FGFR2 Ser24Trp) and MGACA (AKT1 Glu17Lys) [30,31].

## Conclusions

To date, cases of hormone receptor-positive invasive carcinoma arising in association with MGA and/or AMGA are limited to the few case reports described in the literature, as shown in Table 1, which includes salient clinicopathologic data and hormone receptor status. In these cases, it is unclear if MGA is, in fact, a precursor lesion to the synchronous invasive component or is merely a coexisting, unrelated process.

Our SNV analysis indicates an increasing mutational burden with progression from MGA to AMGA to MGACA. The majority (63%) of the SNVs identified in the MGA were retained in the AMGA. Relatively few SNVs are shared by either the MGA (11%) or the AMGA (10%) with the adjacent carcinoma. The considerable overlap between the MGA and AMGA supports the conclusion that the former is a precursor to the latter. This is also consistent with the literature positing a progression from MGA to AMGA through the accumulation of mutations. In this particular case, there is a lack of evidence that either the MGA or the AMGA served as a precursor to the luminal-type MGACA, as the molecular results show a lack of clonal evolution between the entities. A notable finding, however, included the identification of unique oncogenic driver mutations isolated within the AMGA (FGFR2 Ser24Trp) and MGACA (AKT1 Glu17Lys). FGFR2 Ser24Trp is associated with endometrial, breast, ovarian, and cervical cancers (COSMIC database accessed 15 Nov 22). AKT1 Glu17Lys is strongly associated with breast cancer and cited in 374 cases. Interestingly, the FGFR2 Ser24Trp variant has also been previously identified in TP53-mutation negative MGA/AMGA. Mouse studies have demonstrated that this mutation promotes breast cancer by activating the FGFR2/STAT3/MAPK pathway and stimulating the epithelial-to-mesenchymal transition. This missense mutation has also been described within the context of uterine cancer and is one of several oncogenic mutations clustered around the third Ig-like extracellular domain of FGFR2, where they are thought to alter ligand-receptor specificity. The finding of the FGFR2 Ser24Trp variant, a previously characterized oncogenic driver mutation, in this case of MAGA is consistent with AMGA as a precursor lesion for carcinoma, though not in this particular case. AKT1 E17K has been identified in a variety of cancer types, including an estimated 6.7% of ER-positive breast cancers, where it leads to constitutive membrane localization and activation, stimulating survival and proliferation. Taken together, these findings suggest that MGA is the precursor of the AMGA but not of the MGACA in this case, even though they are spatially and temporally related. In the present case, our next-generation sequencing was limited by formalin fixation artifact, precluding copy number variant evaluation, however, future reports would benefit from characterizing copy number variants in hormone receptor-positive MGACA. Documentation of additional cases incorporating molecular information will better elucidate the relationship suggested by this unique case.

## Appendices

Supplementary methods

Case Selection, Histochemistry, and Immunohistochemistry

We selected the case described above for molecular characterization due to the juxtaposition of foci of MGA, AMGA, and luminal-type invasive ductal carcinoma. Two pathologists (one fellowship trained in breast pathology) independently reviewed the hematoxylin and eosin-stained sections. Immunohistochemistry was performed on 4-micron thick formalin-fixed paraffin-embedded (FFPE) sections using the VENTANA BenchMark ULTRA (Roche Diagnostics, Indianapolis, IN). Antigens to the following antibodies were used: Calponin (monoclonal EP798Y, Roche Diagnostics), CD34 (QBEnd/10, Roche Diagnostics), D2-40 (monoclonal D2-40, Roche Diagnostics), ER (monoclonal SP1, Roche Diagnostics), HER2 (monoclonal 4B5, Roche Diagnostics), Ki-67 (monoclonal 30-9, Roche Diagnostics), pancytokeratin (monoclonal Lu-5, Biocare Medical), p53 (monoclonal DO-7, Roche Diagnostics), p63 (monoclonal 4A4, Roche Diagnostics), PR (monoclonal 1E2, Roche Diagnostics), and S100 (polyclonal, Roche Diagnostics). IHC interpretation was performed by a breast fellowship-trained staff pathologist.

Macrodissection and Genomic DNA Extraction

Genomic DNA was extracted from the formalin-fixed, paraffin-embedded normal breast tissue and breast biopsies with representative sections of histologically and immunohistochemically confirmed MGA, AMGA, and hormone receptor-positive invasive ductal carcinoma (3-4, 10µM sections per sample) using the QIAamp DNA FFPE Advanced UNG Kit (50) (Qiagen cat. no. 56704) with Proteinase K lysis, DNA cross-link removal, and RNAse A treatment. The isolated DNA was analyzed via Fragment Analysis using the Agilent High Sensitivity Genomic DNA Kit (Advanced Analytical cat. no. DNF-488-500).

Exome Library Construction

Libraries were constructed using the Illumina® DNA Prep with Enrichment, (S) Tagmentation kit (Illumina cat. no. 20025523). To generate pre-enrichment libraries, genomic DNA was tagmented and cleaned up in Tagmentation Wash Buffer. Tagmented DNA was indexed (IDT Illumina UD indexes set A (Illumina cat. no. 20027213)). Five-hundred (500) ng from each pre-enrichment library were hybridized using probes from the Illumina Exome Panel - Enrichment Oligos Only (Illumina cat. no. 20020183). Hybridized libraries were processed per the Illumina® DNA Prep with Enrichment, (S) Tagmentation kit instructions, and eluted exome-enriched libraries were quantified using the High Sensitivity NGS Fragment Analysis Kit (Advanced Analytical cat. no. DNF-474-0500). Exome-enriched libraries were normalized to 1nM each, pooled, and diluted to a final loading concentration of 180 pM. Pooled libraries were sequenced 2x151 bp using the NovaSeq6000 SP v1.5 kit (Illumina cat. no. 20028400).

De-multiplexed FASTQs were utilized for small nucleotide variant (SNV) analysis using the Illumina DRAGEN Somatic Small Variant Caller in tumor-normal mode, wherein both tumor and normal samples are analyzed jointly to exclude germline variants, generating a tumor-specific VCF (variant call) output file. The mean depth of reading for the normal and tumor samples was 491x and 361 - 429x, respectively. Tumor VCFs were down-sampled to remove variants that failed post-somatic calling filtering steps. The PASS-only VCFs were annotated using the Illumina Nirvana Variant Annotator (https://illumina.github.io/NirvanaDocumentation/). Variants for all samples (2 samples each for MGA, AMGA, and MGACA) were cross-compared using the MOLBIOTOOLS Multiple List Comparator (https://molbiotools.com/listcompare.php) to identify variants shared between the samples.

Supplementary data

**Table 2:** Massively parallel sequence results All single nucleotide variants identified by whole exome sequencing with associated gene, hgsvg, coding change, protein change, and variant allele frequency identified in microglandular adenosis (MGA), atypical microglandular adenosis (AMGA), and microglandular associated carcinoma (MGACA)

Gene	HGVSg	Coding Change	Protein Change	MGA	AMGA	MGACA
CNMD	NC_000013.11:g.52708664C>A	c.661G>T	p.(Val221Phe)	0.31	0.14	
WFDC1	NC_000016.10:g.84318334G>A	c.400G>A	p.(Gly134Ser)	0.30	0.17	
INPP5K	NC_000017.11:g.1495875G>A	c.1295C>T	p.(Pro432Leu)	0.26	0.22	
SBF1	NC_000022.g.50460637G>A11:	c.3043C>T	p.(Arg1015Trp)	0.22	0.20	
PKHD1L1	NC_000008.11:g.109442073G>T	c.4271G>T	p.(Trp1424Leu)	0.13	0.20	
EIF1AX	NC_000023.11:g.20138614C>G	c.25G>C	p.(Gly9Arg)	0.19	0.15	
SRRM2	NC_000016.10:g.2758547G>A	c.593G>A	p.(Arg198His)	0.31	0.22	
FOXN1	NC_000017.11:g.28534430_28534468dup	c.1027_1065dup	p.(Gly343_Lys355dup)		0.26	
CHD3	NC_000017.11:g.7910509C>T	c.5849C>T	(Pro1950Leu)	0.19	0.18	
CTIF	NC_000018.10:g.48758217G>A	c.883G>A	p.(Glu295Lys)	0.27	0.28	
RYR2	NC_000001.11:g.237649917G>C	c.7553G>C	p.(Arg2518Pro)		0.20	
TRPM5	NC_000011.10:g.2414093C>T	c.1840G>A	p.(Ala614Thr)		0.26	
ALG1	NC_000016.10:g.5079075T>G	c.541T>G	p.(Phe181Val)	0.02		0.17
JAM2	NC_000021.9:g.25714267A>G	c.901A>G	p.(Lys301Glu)	0.04		
EAF1-AS1	NC_000003.12:g.15458289A>T	c.821T>A	p.(Phe274Tyr)		0.17	
EPHB3	NC_000003.12:g.184580443_184580454del	c.2214_2225del	p.(Met738_Gly741del)		0.22	
NSA2	NC_000005.10:g.74773929C>T	c.584C>T	p.(Pro195Leu)		0.21	
PAX5	NC_000009.12:g.36840609G>T	c.1127C>A	p.(Ala376Asp)		0.22	
WWC3	NC_000023.11:g.10098539C>T	c.691C>T	p.(Arg231Ter)		0.28	
CASZ1	NC_000001.11:g.10645076G>A	c.3709C>T	p.(Arg1237Ter)		0.03	
LEFTY2	NC_000001.11:g.225937684del	c.758del	p.(Gly253AlafsTer40)			0.48
EGLN1	NC_000001.11:g.231367621_231367622del	c.1164_1165del	p.(Trp389ValfsTer3)			0.01
ARID1A	NC_000001.11:g.26780451dup	c.5401dup	p.(Ile1801AsnfsTer40)		0.20	
FGFR2	NC_000010.11:g.121520163G>C	c.755C>G	p.(Ser252Trp)		0.23	
RHOBTB1	NC_000010.11:g.60886167C>T	c.1520G>A	p.(Cys507Tyr)			0.10
GATA3	NC_000010.11:g.8064095T>G	c.881T>G	p.(Met294Arg)			0.21
HELLS	NC_000010.11:g.94607972A>G	c.880A>G	p.(Thr294Ala)	0.04		
SIAE	NC_000011.10:g.124660696G>A	c.337C>T	p.(His113Tyr)			0.27
OR51A4	NC_000011.10:g.4946865G>A	c.236C>T	p.(Ser79Phe)		0.04	
PRB3	NC_000012.12:g.11267347C>T	c.776G>A	p.(Arg259His)			0.18
DDX47	NC_000012.12:g.12827328A>T	c.1042A>T	p.(Met348Leu)			0.06
AKT1	NC_000014.9:g.104780214C>T	c.49G>A	p.(Glu17Lys)			0.24
GOLGA8T	NC_000015.10:g.30144869C>T	c.1459C>T	p.(Arg487Cys)		0.18	
KATNBL1	NC_000015.10:g.34143000T>A	c.224A>T	p.(Arg76Trp)		0.07	
ACSF3	NC_000016.10:g.89154104C>G	c.1628C>G	p.(Pro543Arg)		0.14	
FANCA	NC_000016.10:g.89778402C>G	c.261G>C	p.(Lys87Asn)		0.09	
KCNH4	NC_000017.11:g.42165657G>A	c.1877C>T	p.(Pro626Leu)		0.03	0.03
EVPL	NC_000017.11:g.76009234G>A	c.3971C>T	p.(Pro1324Leu)		0.15	
QRICH2	NC_000017.11:g.76277294C>T	c.4636G>A	p.( Asp1546Asn)			0.15
TBCD	NC_000017.11:g.82831928del	c.528del	p.(Glu177ArgfsTer189)		0.28	
KMT2B	NC_000019.10:g.35733369C>	c.6820C>T	p.(Gln2274Ter)			0.28
PPRG	NC_000019.10:g.49588524_49588525insCCCAGGCCGGCCAGGACAATGAGCAGGATGCCACCTGTCAGCCCCACAGCCA	c.329_330insCCCAGGCCGGCCAGGACAATGAGCAGGATGCCACCTGTCAGCCCCACAGCCA	p.(NOSIP Ala111ProfsTer7)			0.03
DYSF	NC_000002.12:g.71574295G>T	c.3365G>T	(Arg1122Leu)	0.04		0.03
BSN	NC_000003.12:g.49657459C>T	c.7903C>T	p.(Arg2635Cys)			0.23
ATP10D	NC_000004.12:g.47546689G>A	c.1462G>A	p.(Gly488Ser)			0.18
PCDHB9	NC_000005.10:g.141189429C>T	c.2111C>T	p.(Ser704Leu)			0.10
SPINK5	NC_000005.10:g.148124790A>C	c.2692A>C	p.(Lys898Gln)			0.15
CPEB4	NC_000005.10:g.173889854C>A	c.121C>A	p.(Pro41Thr)			0.00
RACK1	NC_000005.10:g.181237713C>T	c.136G>A	p.(Glu46Lys)			0.32
NHSL1	NC_000006.12:g.138433015A>C	c.1330T>G	p.(Ser444Ala)		0.01	
LPA	NC_000006.12:g.160556082G>T	c.4916C>A	p.(Ser1639Tyr)			0.25
MDGA1	NC_000006.12:g.37652038C>T	c.1285G>A	p.(Val429Ile)			0.20
CD2AP	NC_000006.12:g.47503342G>A	c.67G>A	p.(Gly23Arg)			0.15
FAM83B	NC_000006.12:g.54941656A>T	c.2685A>T	p.(Gln895His)			0.24
EPHA7	NC_000006.12:g.93269559_93269560insACCAAGTCTACTTCA	c.1551_1552insGAAGTAGACTTGGTT	p.(Ala517_Phe518insGluValAspLeuVal)		0.03	
EPHA7	NC_000006.12:g.93269562_93269563insT	c.1547_1548insA	p.(Ala517GlyfsTer15)		0.03	
EPHA7	NC_000006.12:g.93269563_93269564insCATTAA	c.1546_1547insTTAATG	p.(Arg516delinsLeuAsnGly)		0.03	
OPLAH	NC_000008.11:g.144054625_144054630del	c.2617_2622del	p.(Met873_Leu874del)			0.33
KIAA2026	NC_000009.12:g.5968179C>A	c.2052G>T	p.(Lys684Asn)			0.22
TMEM185A	X-149611352-C-A	c.150G>T	p.(Trp50Cys)		0.08	
F8	NC_000023.11:g.154993032C>T	c.505G>A	p.(Asp169Asn)			0.11
GART	NC_000021.9:g.33529739A>G	c.389T>C	p.(His130=)		0.19	0.09
GDPD5	NC_000011.10:g.75441262T>A	c.960A>T	p.(Ala320=)	0.23	0.17	
GPR108	NC_000019.10:g.6733221G>A	c.804C>T	p.(Ser268=)	0.22	0.19	
TTN	NC_000002.12:g.178629307G>A	c.36714C>T	p.(Ser12238=)	0.27	0.17	
AQP12B	NC_000002.12:g.240682778T>C	c.60A>G	p.(Ala20=)			0.18
TMEM230	NC_000020.11:g.5111587C>T	c.87G>A	p.(Ser29=)		0.06	0.05
FSTL4	NC_000005.10:g.133400793A>T	c.354T>A	p.(Ala118=)			0.04
PLXNA3	NC_000023.11:g.154460435C>T	c.252C>T	p.(Arg84=)		0.44	
CSMD2	NC_000001.11:g.33586610G>A	c.6945C>T	p.(Ile2315=)			0.02
CES4A	NC_000016.10:g.67001401A>G	c.630A>G	p.(Gly210=)		0.16	
SYNRG	NC_000017.11:g.37584664C>T	c.573G>A	p.(Ser191=)	0.02		
ZFP36	NC_000019.10:g.39408066G>A	c.366G>A	p.(Gly122=)			0.22
VN1R2	NC_000019.10:g.53258408T>G	c.33T>G	p.(Ala11=)			0.19
MLLT1	NC_000019.10:g.6270631G>A	c.141C>T	p.(Phe47=)			0.21
MARCH 2	NC_000019.10:g.8438420C>T	c.405C>T	p.(Ser135=)			0.02
MAP2	NC_000002.12:g.209693460A>G	c.1290A>G	p.(Gly430=)			0.09
RNPEPL1	NC_000002.12:g.240569027C>T	c.441C>T	p.(Ser147=)		0.26	
STOX2	NC_000004.12:g.184011268G>A	c.2430G>A	p.(Lys810=)			0.17
PDGFRB	NC_000005.10:g.150121926G>A	c.2298C>T	p.(Ile766=)			0.19
NOS3	NC_000007.14:g.150993950G>A	c.147G>A	p.(Ala49=)			0.17
FOXK1	NC_000007.14:g.4754573G>A	c.861G>A	p.(Ala287=)			0.01
CUL4B	NC_000023.11:g.120560501A>G	c.138T>C	p.(Ser46=)			0.09
MAP7D2	NC_000023.11:g.20025775C>T	c.1062G>A	p.(Pro354=)	0.01		0.02
MIR1304	NC_000011.10:g.93733692G>C	n.73C>G		0.26	0.27	0.31
TAF1D	NC_000011.10:g.93733697C>T	c.*57-300G>A		0.28	0.30	0.44
PAX2	NC_000010.11:g.100825007C>T	c.1114+258C>T		0.25	0.17	
KRAS	NC_000012.12:g.25227447G>A	c.112-35C>T		0.25	0.12	
SH2B2	NC_000007.14:g.102309621G>A	c.1052+715G>A				0.09
AC009264.1	NC_000007.14:g.137014982C>T	n.273+17812G>A		0.31	0.25	
C9orf3	NC_000009.12:g.95085520T>C	c.*5-1162T>C		0.05	0.04	0.05
COQ5	NC_000012.12:g.120523976T>C	c.203-1613A>G				0.05
BBOF1	NC_000014.9:g.74054870C>G	c.1287-714C>G			0.15	0.08
SENP3-EIF4A1	NC_000017.11:g.7576828A>G	n.3076+136A>G			0.20	
PRKCG	NC_000019.10:g.53900404del	c.1374-15del			0.02	
DOCK3	NC_000003.12:g.50880591C>T	c.163-9435C>T			0.19	0.19
AC108860.2	NC_000008.11:g.94639269G>C	n.199C>G			0.25	
ANKS6	NC_000009.12:g.98768252T>A	c.378-2A>T			0.02	0.03
PPP2R3B	NC_000023.11:g.345123T>C	c.1036+393A>G			0.20	
MDS2	NC_000001.11:g.23628522G>A	n.1333G>A			0.09	
PLD5	NC_000001.11:g.242265338del	c.607+1del			0.03	
EFCAB2	NC_000001.11:g.245015681A>G	n.95-1561A>G				0.06
PABPC4	NC_000001.11:g.39571626T>C	c.388-277A>G				0.06
TCEANC2	NC_000001.11:g.54104672A>G	n.1086-6380A>G				0.06
C8A	NC_000001.11:g.56876215G>A	c.464+6G>A				0.14
PARGP1	NC_000010.11:g.45900396C>T	n.1952G>A			0.13	
RNLS	NC_000010.11:g.88545515C>T	c.526+27388G>A			0.01	0.05
HECTD2	NC_000010.11:g.91482992C>T	n.426+42036G>A			0.02	0.02
CYP2C19	NC_000010.11:g.94762701_94762728del	c.-5_23del			0.05	
NDUFA9	NC_000012.12:g.4667549A>T	c.656-908A>T		0.10		0.07
TBC1D15	NC_000012.12:g.71894850A>G	c.822A>G	p.(TBC1D15)			0.21
NUBPL	NC_000014.9:g.31562858_31562859insC	c.256+643_256+644insC				0.17
BUB1B	NC_000015.10:g.40166445A>T	c.179+1249A>T				0.11
ETFA	NC_000015.10:g.76284861T>C	c.664+776A>G				0.07
ETFA	NC_000015.10:g.76284861_76284862inv	c.664+775_664+776inv				0.07
ETFA	NC_000015.10:g.76284862G>A	c.664+775C>T				0.07
ICAM1	NC_000019.10:g.10283747T>G	n.141+1221A>C				0.05
MUM1	NC_000019.10:g.1360218C>T	n.1877C>T				0.20
GATAD2A	NC_000019.10:g.19441611A>G	c.-7+5392A>G				0.06
HAMP	NC_000019.10:g.35284904C>T	c.151-34C>T			0.20	
RAB3GAP1	NC_000002.12:g.135089783T>C	c.151-1215T>C				0.04
SAG	NC_000002.12:g.233338850_233338858del	c.1022+97_1022+105del				0.04
SAG	NC_000002.12:g.233338861_233338864del	c.1022+108_1022+111del				0.04
SAG	NC_000002.12:g.233338868_233338872del	c.1022+115_1022+119del				0.04
FAM228B	NC_000002.12:g.24082973G>A	n.167+2018G>A				0.15
ATP6V1E1	NC_000022.11:g.17619083A>G	c.99+378T>C		0.05		
IFT122	NC_000003.12:g.129457945G>A	c.-257-654G>A			0.10	
GPR78	NC_000004.12:g.8603962dup	c.*405-14dup				0.19
AP002026.1	NC_000004.12:g.99099909T>G	n.428+10625T>G				0.04
ARL10	NC_000005.10:g.176367968C>A	c.386-839C>A				0.04
FAM184A	NC_000006.12:g.119069062A>G	c.30+9079T>C		0.06		
F13A1	NC_000006.12:g.6222178G>A	c.974-7C>T				0.16
STYXL1	NC_000007.14:g.75999116T>C	n.699-2517A>G			0.07	
FGFR1	NC_000008.11:g.38414061G>A	c.2280-38C>T				0.15
ADAM32	NC_000008.11:g.39131948T>C	c.232-4709T>C				0.09
ANXA2P2	NC_000009.12:g.33624963_33624964insCC	n.739_740insCC			0.02	
